# Whole Genome Sequence Analysis of *Cryptococcus gattii* from the Pacific Northwest Reveals Unexpected Diversity

**DOI:** 10.1371/journal.pone.0028550

**Published:** 2011-12-07

**Authors:** John D. Gillece, James M. Schupp, S. Arunmozhi Balajee, Julie Harris, Talima Pearson, Yongpan Yan, Paul Keim, Emilio DeBess, Nicola Marsden-Haug, Ron Wohrle, David M. Engelthaler, Shawn R. Lockhart

**Affiliations:** 1 Pathogen Genomics Division, Translational Genomics Research Institute, Flagstaff, Arizona, United States of America; 2 Mycotic Diseases Branch, Centers for Disease Control and Prevention, Atlanta, Georgia, United States of America; 3 Center for Microbial Genetics and Genomics, Northern Arizona University, Flagstaff, Arizona, United States of America; 4 Center for Genetic Epidemiology and Prevention, Van Andel Institute, Grand Rapids, Michigan, United States of America; 5 Oregon State Department of Human Services, Portland, Oregon, United States of America; 6 Washington State Department of Health, Shoreline, Washington, United States of America; 7 Washington State Department of Health, Tumwater, Washington, United States of America; University of Sydney, Australia

## Abstract

A recent emergence of *Cryptococcus gattii* in the Pacific Northwest involves strains that fall into three primarily clonal molecular subtypes: VGIIa, VGIIb and VGIIc. Multilocus sequence typing (MLST) and variable number tandem repeat analysis appear to identify little diversity within these molecular subtypes. Given the apparent expansion of these subtypes into new geographic areas and their ability to cause disease in immunocompetent individuals, differentiation of isolates belonging to these subtypes could be very important from a public health perspective. We used whole genome sequence typing (WGST) to perform fine-scale phylogenetic analysis on 20 *C. gattii* isolates, 18 of which are from the VGII molecular type largely responsible for the Pacific Northwest emergence. Analysis both including and excluding (289,586 SNPs and 56,845 SNPs, respectively) molecular types VGI and VGIII isolates resulted in phylogenetic reconstructions consistent, for the most part, with MLST analysis but with far greater resolution among isolates. The WGST analysis presented here resulted in identification of over 100 SNPs among eight VGIIc isolates as well as unique genotypes for each of the VGIIa, VGIIb and VGIIc isolates. Similar levels of genetic diversity were found within each of the molecular subtype isolates, despite the fact that the VGIIb clade is thought to have emerged much earlier. The analysis presented here is the first multi-genome WGST study to focus on the *C. gattii* molecular subtypes involved in the Pacific Northwest emergence and describes the tools that will further our understanding of this emerging pathogen.

## Introduction

*Cryptococcus gattii* is an emerging cause of community-acquired fungal pneumonia, meningitis, and disseminated disease. In the past decade, infections caused by *C. gattii* have been increasingly identified outside of the previously recognized tropical and subtropical endemic regions and have emerged in temperate regions, including British Columbia (BC), the US Pacific Northwest (PNW), and Japan [1]–[4].

Emergence of *C. gattii* in BC and the PNW is remarkable for a number of reasons, including the adaptation of the pathogen to a climate previously unrecognized as potentially hospitable, an apparent increased virulence of certain molecular subtypes in mouse models [5], and the identification of novel clonal molecular subtypes [6]–[8]. Veterinary cases were first identified in 2000 on Vancouver Island, BC [9], and subsequent surveillance identified human cases beginning in 1999 on Vancouver Island and in 2004 in the US PNW [2], [3]. By 2011, cases from this emergence had been identified on Vancouver Island, the BC mainland, and in the states of Washington, Oregon, Idaho and California in the US.

Strains of *C. gattii* have been differentiated through the use of restriction fragment length polymorphism (RFLP), amplified fragment length polymorphism (AFLP), and multi-locus sequence typing (MLST) into four main molecular types: VGI, VGII, VGIII and VGIV. Further work using MLST has determined that these groups are monophyletic and genotypically distinct enough to deserve varietal status [10], [11]. While there is genetic diversity within each of the *C. gattii* molecular types, a unique feature of the *C. gattii* emergence in BC and the PNW is the distinct clonality of these isolates. The predominant *C. gattii* molecular type VGII sorts into three closely related clonal subtypes designated VGIIa, VGIIb, and VGIIc [6], [7], [12], [13]. The major subtype in BC was designated VGIIa because it was the predominant subtype and it had not been previously recognized from global collections [6], [7]. The minor subtype in BC was designated VGIIb, and has been previously identified from other parts of the world, which led to speculation that it may be the progenitor of subtype VGIIa [7], [14]. The strains identified in Oregon and Washington include VGIIa, VGIIb and a unique VGII subtype not identified in BC and has since been designated VGIIc [12]. VGIIc shares a number of MLST alleles with both VGIIa and VGIIb and has novel MLST alleles as well [12], [13]. These novel VGIIc strains were highly virulent in a murine pulmonary model of infection and had similar virulence features to VGIIa strains studied from Vancouver Island [5]. Isolates belonging to the VGIIc subtype group have not been identified outside of the PNW.

While MLST has been able to distinguish adequately between the VGIIa, VGIIb and VGIIc subtypes within the BC and PNW emergence, neither MLST nor variable number tandem repeat analysis (VNTR) have been successful at distinguishing among isolates within each clonal group. To date, only one single nucleotide polymorphism (SNP) has been identified within the VGIIc clonal group, and one VNTR difference has been identified within the VGIIa clonal group using these methods [5], [13]. Like most typing systems, MLST and VNTR are limited by the small portion of the genome that is surveyed. Whole Genome Sequence Typing (WGST) is a methodology that maximizes the data available for inference of genetic diversity and has been successfully used to distinguish among highly related isolates of the fungus *Coccidioides immitis* [15]. Here we describe the WGST analysis of *C. gattii* using isolates from the PNW emergence. This next generation typing methodology allowed us to generate a unique genetic fingerprint for each study isolate, effectively subtyping a “clonal” population.

## Materials and Methods

### Isolates and DNA extraction

*C. gattii* isolates were received at the Centers for Disease Control and Prevention from state and local health departments, clinicians, veterinarians and ongoing environmental studies (Table 1) in the PNW US and BC. Isolates were grown on YPD plus 0.5% NaCl and DNA was prepared using an UltraClean DNA Isolation Kit as described by the manufacturer (MO BIO Laboratories, Carlsbad, CA). Twenty *C. gattii* isolates representing three of the four previously described molecular types (VGI, VGII, and VGIII) and two published *C. gattii* genomes [16] were used in the analysis. The majority of the isolates (n = 18) were known to belong to the VGIIa (6 isolates), VGIIb (4 isolates) or VGIIc (8 isolates) molecular subtypes, with an additional one each from the VGI and VGIII types. All isolates were from the PNW and BC emergence.

**Table 1:** 

MLST Type	Strain ID	Geographic Source	Clinical Source	Collection Date	Reference Genome covered at ≥10× (%)	Average Read Depth
**IIa**	B7395	Washington	Dog	2008	96.79	39
	B7467	Oregon	Porpoise	2009	96.69	39
	B8849	Oregon	Environmental	2010	96.93	57
	B8577	British Columbia	Environmental	2009	96.71	42
**IIb**	B7422	Oregon	Cat	2009	97.41	94
	B7436	N. California	Alpaca	2009	97.47	108
	B7394	Washington	Cat	2008	86.65	18
	B7735	Oregon	Human	2009	87.00	19
	B8554	Oregon	Dog	2008	95.82	70
	B8828	Washington	Porpoise	2010	93.72	34
**IIc**	B8571	Washington	Human	2009	95.06	46
	B8843	Oregon	Human	2010	95.26	58
	B8838	Washington	Human	2010	95.19	55
	B7466	Oregon	Cat	2008	94.76	42
	B7737	Oregon	Human	2009	94.74	39
	B6863	Oregon	Human	2005	94.09	32
	B7390	Idaho	Human	2008	93.69	30
	B7432	Oregon	Human	2009	92.90	25
**I**	B7488	Oregon	Human	2009	72.71	33
**III**	B8212	Oregon	Human	2007	64.25	16

### MLST analysis

MLST gene fragments were amplified using the seven consensus primers of Meyer et al. [17] as previously described [13]. All loci were sequenced using BigDye terminator technology (ABI, Foster City, CA) with an ABI Prism 3730 DNA sequencer in both forward and reverse directions with the same primers as those used for the PCR reactions.

### Whole Genome Sequencing

The DNA samples were prepared for multiplexed, paired-end sequencing on the Illumina GAIIx Genome Analyzer (Illumina, Inc, San Diego, CA). For each isolate, 1–5 µg dsDNA in 200 µl was sheared in a 96-well plate with the SonicMAN™ (Part # SCM1000-3, Matrical Bioscience, Spokane, WA) to a size range of 200–1000 base pairs with the majority of material at ca. 600 base pairs using the following parameters: Pre Chill - 0°C for 75 sec; Cycles - 20; Sonication - 10 sec; Power - 100%; Lid Chill - 0°C for 75 sec; Plate Chill - 0°C for 10 sec; Post Chill - 0°C for 75 sec. The sheared DNA was purified using the QIAquick PCR Purification kit (Cat #28106, Qiagen,Valencia, CA). The enzymatic processing (end-repair, phosphorylation, A-tailing, and adaptor ligation) of the DNA followed the guidelines as described in the Illumina protocol (“Preparing Samples for Multiplexed Paired-End Sequencing”, Catalog #PE-930-1002, Part #1005361). The enzymes for processing were obtained from New England Biolabs (Cat #E6000L, New England Biolabs, Ipswich, MA) and the oligonucleotides and adaptors were obtained from Illumina (Cat #PE-400-1001). After ligation of the adaptors, the DNA was run on a 2% agarose gel for 2 hours, after which a gel slice containing 500–600 bp fragments of each DNA sample was isolated and purified using the QIAquick Gel Extraction kit (Cat #28706, Qiagen, Valencia, CA). Individual libraries were quantified with qPCR on the ABI 7900HT (Part #4329001, Life Technologies Corporation, Carlsbad, CA) in triplicate at two concentrations, 1∶1000 and 1∶2000, using the Kapa Library Quantification Kit (part # KK4832 or KK4835, Kapa Biosystems, Woburn, MA). Based on the individual library concentrations, equimolar pools of no more than 5 indexed *Cryptococcus* libraries were prepared at a concentration of at least 1 nM using 10 mM Tris-HCl, pH 8.0+0.05% Tween 20 as the diluent. To ensure accurate loading onto the flowcell, the same quantification method was used to quantify the final pools. The pooled libraries were sequenced on the Illumina GAIIx. A 75 bp read paired end run was used for the first 2 isolates sequenced; the remaining isolates were done with a 101 bp read paired end run.

### MLST and WGST Sequence Analysis

The seven MLST gene fragment sequences were concatenated, aligned, and subjected to maximum parsimony analysis using MEGA4 [18].

Illumina WGS data sets were aligned against the *Cryptococcus neoformans* var *gattii* R265 genome [19] (molecular subtype VGIIa) using the short-read alignment component of the Burrows-Wheeler Aligner [20].

Reads containing insertions or deletions, and those mapping to multiple locations in the reference were removed from the final alignments. Each alignment was analyzed for SNPs using SolSNP (http://sourceforge.net/projects/solsnp/). SNPs were excluded if they did not meet a minimum coverage of 10× and if the variant was present in less than 90% of the base calls for that position. In parallel, publically available genomes were aligned against R265 using MUMmer 3.22. SNPs were extracted from the alignments using a custom script. Subsequently, regions found to be duplicated with the R265 reference genome were identified using MUMmer version 3.22 [21]. SNPs residing within these repetitive regions were then removed. Loci that lacked reference sequence coverage data for one or more isolates were removed from the final analysis. The remaining loci were compiled in a matrix of orthologous SNPs loci shared across all genomes. Phylogenetic analyses were performed using the maximum parsimony algorithm, default parameters, in MEGA5 [18]. Where shown, 1000 generations were run for bootstrap analysis. Reference genome mapping and read depth statistics were determined using Lasergene's Seqman NGEN™ V. 2.2 software (Lasergene, Madison, WI). The Illumina WGS data files have been deposited in the NCBI Sequence Read Archive (http://www.ncbi.nlm.nih.gov/sra, accession # SRP006436).

## Results

**Figure 1:**
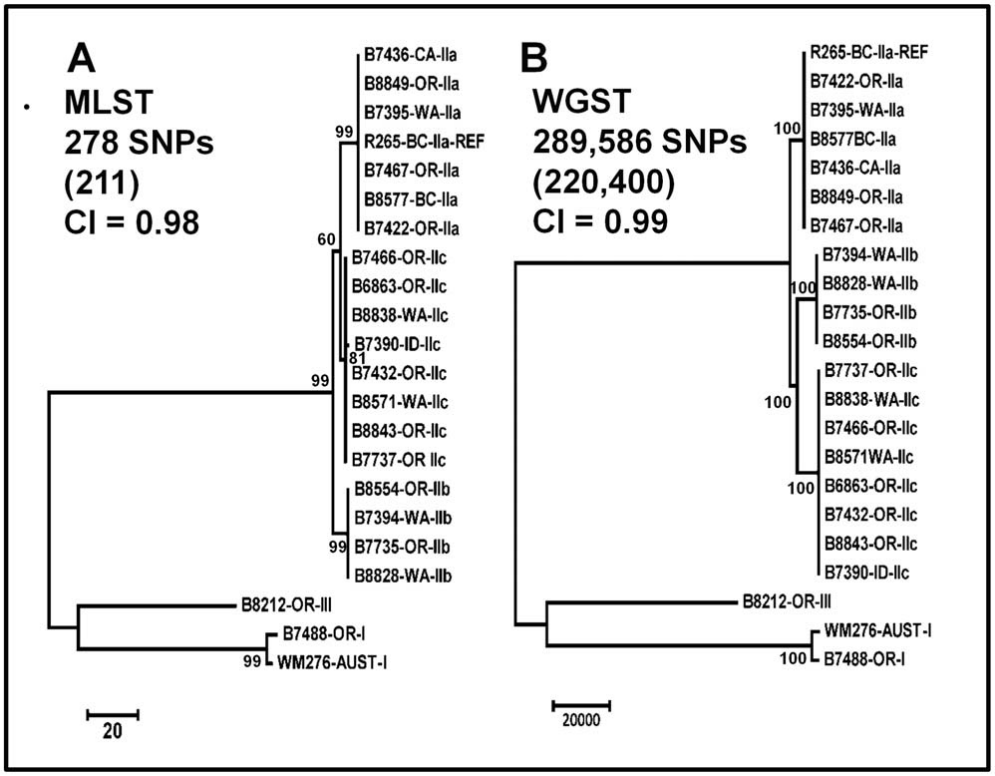
Phylogenetic analysis comparison of MLST and WGST data from 22 *C. gattii* isolates. Maximum parsimony phylogenetic analysis was performed in MEGA4 on 22 *C. gattii* genomes with MLST (panel A, one of 270 most parsimonious trees shown) and WGST (panel B, one of 190 most parsimonious trees shown) data. The R265 whole genome sequence was used as the reference for SNP discovery. Both trees are rooted on the VGI/VGIII branch. Bootstrap values less than 50% and for intra-VGIIa, VGIIb and VGIIc branches are not shown. The taxa nomenclature includes a four digit unique identifier, the location of origin (BC = British Columbia, CA = California, ID = Idaho, OR = Oregon and WA = Washington state), and the molecular subtype as determined by MLST analysis (I, IIa, IIb, IIc and III). The number of SNPs included in each data set is indicated (number of parsimony-informative SNPs in parentheses), as is the consistency index (CI) as calculated by MEGA4. While WGST analysis found unique genotypes for all isolates, they are not visible on this tree due to the large numbers of SNPs separating the VGII from VGI and VGIII isolates.

Twenty two *C. gattii* genomes representing three of the four previously described molecular types (VGI, VGII, and VGIII) were subjected to MLST analysis using the consensus seven loci [17]. MLST analysis delineated 278 single nucleotide polymorphisms (SNPs) among the 22 *C. gattii* genomes, 211 of which were parsimonious, with the majority being among the different subtypes (Figure 1). Among the three VGII subtypes, MLST was able to distinguish 18 SNPs, 11 of which were parsimonious. Within each of the VGII subtypes all genomes were clonal with the exception of a single non-parsimonious SNP among the VGIIc isolates.

**Figure 2:**
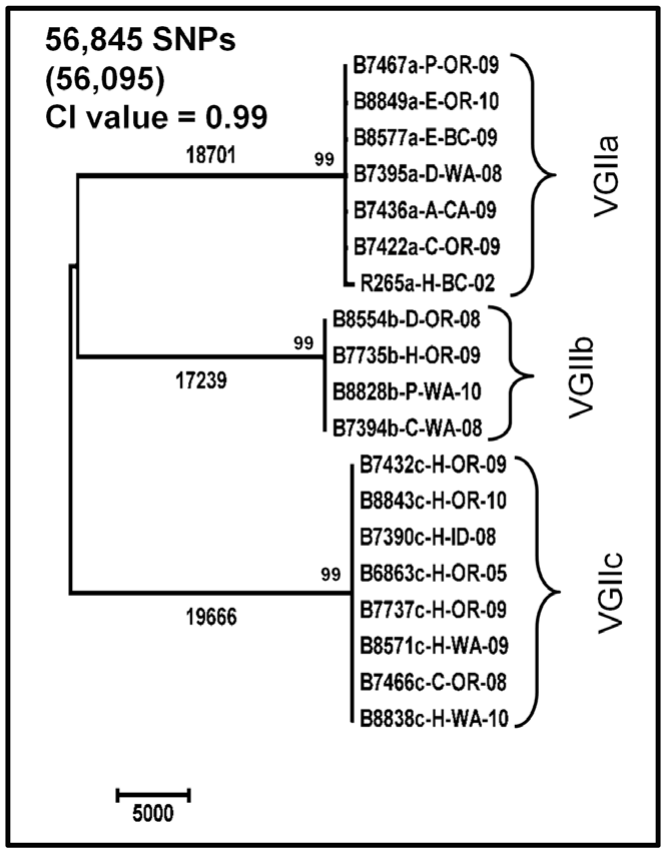
Phylogenetic analysis of WGST data from *C. gattii* VGII molecular type isolates. Maximum parsimony phylogenetic analysis was performed in MEGA4 on WGST SNP data from 18 VGII *C. gattii* genomes using the R265 whole genome sequence as the reference for SNP discovery (R265a-H-BC-02). The analysis found 76 most parsimonious trees, one of which is shown. The total number of SNPs included in the analysis is indicated (number of parsimonious SNPs in parentheses) as is the consistency index (CI) as calculated by MEGA4. Bootstrap values less than 50% are not shown. The tree shown is rooted on the mid-point. Branch lengths as calculated by MEGA4 are indicated above the three major branches. All other branch lengths are less than 500. The taxa nomenclature include a unique four digit identifier, the molecular subtype (a, b or c), the source of the isolate (A = alpaca, C = cat, D = dog, E = environmental, H = human and P = porpoise), the location of origin (BC = British Columbia, CA = California, ID = Idaho, OR = Oregon and WA = Washington state), and the year of collection. For example, B7395a-D-WA-08 indicates that isolate B7395 is a VGIIa subtype collected from a dog in Washington state in 2008.

**Figure 3:**
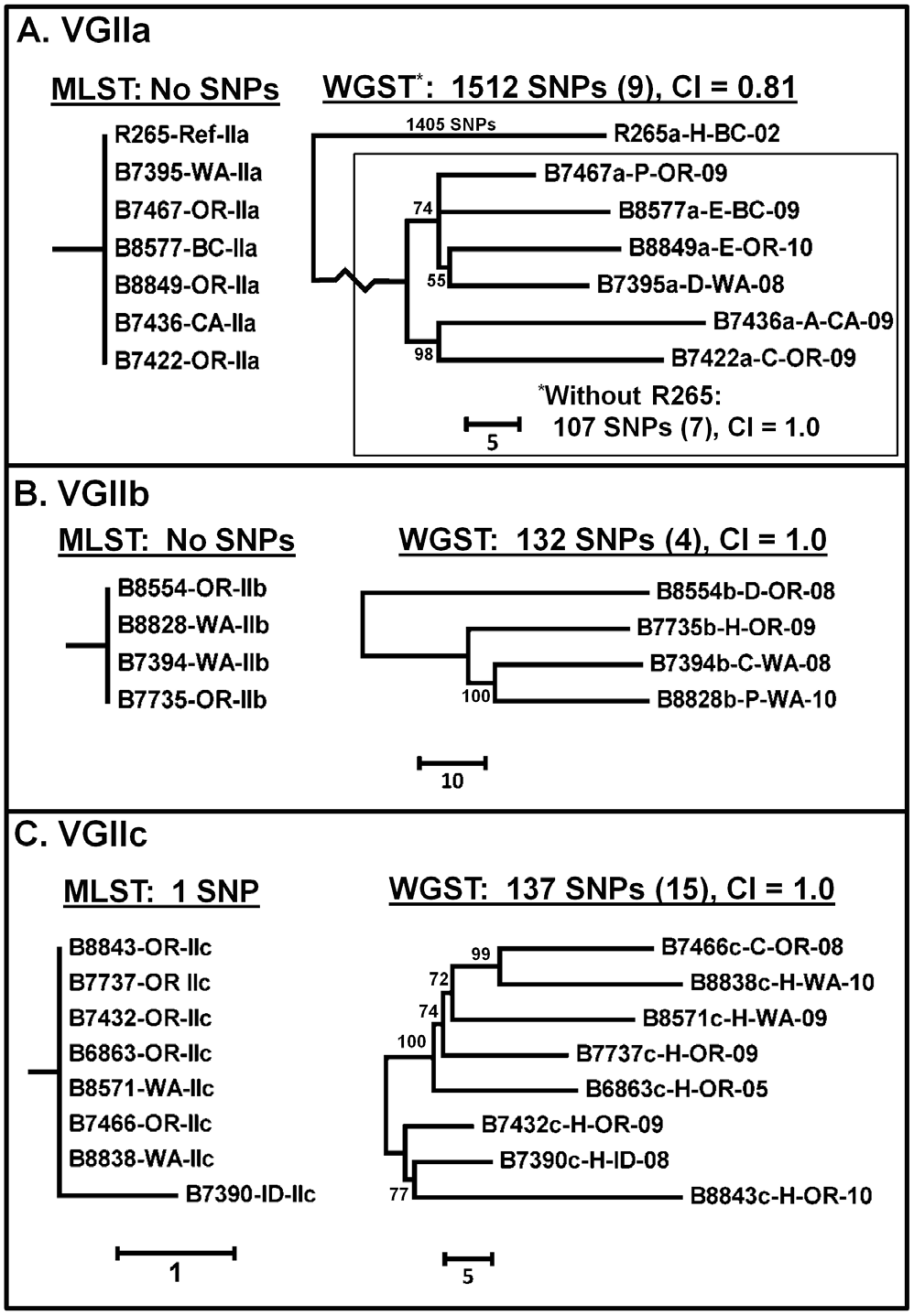
Comparison of MLST and WGST analysis of VGIIa, VGIIb and VGIIc *C. gattii* isolates. Maximum parsimony phylogenetic analysis was performed in MEGA4 on MLST and WGST SNP data from VGIIa (panel A), VGIIb (panel B) and VGIIc (panel C) genomes. Scale bars indicate relative branch lengths for each analysis. The R265 whole genome sequence was used as the reference for SNP discovery (R265a-H-BC-02). VGIIa (panel A) analysis is shown both with and without R265, VGIIb and VGIIc shown only without R265. The numbers of SNPs included in each analysis is indicated. The consistency index value (CI) was calculated in MEGA4. Meaning of taxa nomenclature is described in legends for Figures 1 (MLST) and 2 (WGST). Trees shown are mid-point rooted, bootstrap values less than 50% are not shown.

The same set of 22 genomes was analyzed by WGST analysis (Figure 1). The average read depth of the VGII isolates, when aligned to the R265 reference genome (VGIIa, 17.5 Mbp), ranged from18–108×, with reads from all VGII isolates mapping to >86% of the reference genome. The VGI and VGIII genomes had average read depths of 33× and 16× with reference genome coverage of 72.7% and 64.2%, respectively. The average distance between paired reads was 450 bp. Among all 22 genomes, 289,586 SNPs were identified, 220,400 (76.1%) of which were parsimony-informative. There were a small number of insertion and deletion differences between VGI/VGIII and VGII, but none among the VGII genomes. These insertions and deletions were not incorporated into the phylogenetic analysis. The trees shown were rooted with VGI and VGIII genomes as we established that they formed an outgroup to the VGII group (Figure S1). When the VGI and VGIII genomes were excluded, WGST analysis found 56,845 SNPs, 56,095 (98.7%) of which were parsimony-informative, among all three VGII subtypes (Figure 2). Excluding more distantly related genomes from the WGS SNP discovery analysis increases the portion of the reference genome shared and, therefore, the number of shared SNP loci found among the remaining genomes. Branch lengths for the VGIIa, VGIIb, and VGIIc subclades were 18,701, 17,239, and 19,666 SNPs, respectively. All other branch lengths on this tree were less than 500 SNPs. The Consistency Index (CI) for the VGII isolate Maximum Parsimony analysis was 0.99. Separate analyses on each of the VGII subtypes found a single most parsimonious tree for each subtype and 1,512 SNPs (9 (0.6%) parsimony-informative), 132 SNPs (4 (3%) parsimony-informative) and 137 SNPs (15 (10.9%) parsimony-informative) within the VGIIa, VGIIb and VGIIc subtypes, respectively (Figure 3). Within-subtype analyses resulted in similarly high CI values, indicating minimal homoplasy within these data sets.

The phylogenetic relationships among the various subtypes and general topology of the trees were consistent, for the most part, between MLST and WGST analysis, both providing clear separation of each subtype (Figure 1). The major difference between the two types of analysis was the improved resolving power of WGST over MLST within each of the VGII clonal subtypes. Only a single SNP difference was found within any of the subtypes (VGIIc) using MLST, whereas unique genotypes were generated for each isolate by WGST.

## Discussion

Employing next generation sequencing to analyze a large number of *C. gattii* genomes (1) demonstrate that WGST has superior resolving power as compared to available sub-typing tools; (2) reveals the presence of unique genotypes within VGII “clonal” subtypes; and (3) yields evidence that while the VGIIb sublineage may have radiated earlier than the other two sublineages, it may not have given rise to them.

In the present study, *C. gattii* WGST analysis identified a substantial number of genomic differences that were undetectable by the currently available typing methodologies that include MLST and VNTR analysis [5]. Analyses of twenty two genomes by both MLST and WGST revealed that while the former method generated only 18 SNPS, the latter method revealed nearly 57,000 SNPs that discriminated among and within the three VGII molecular subtypes. Further, WGST analysis revealed over one hundred SNPs within each VGII clonal subtype, more than MLST revealed across all VGII subtypes. More importantly, MLST analysis revealed no SNPs among the VGIIa and VGIIb subtypes and only one SNP among the VGIIc isolates, leaving individual isolates undifferentiated, while WGST analysis was able to generate 22 unique genotypes among the 22 genomes analyzed in addition to establishing the phylogenetic structure among all isolates and defining fine scale evolutionary relationships between the individual isolates.

Of note is the observation that most of the SNPs in VGIIa are found on the branch leading to the single R265 genome; when this genome is removed from the analysis, only 107 SNPs are identified among the remaining VGIIa isolates, seven of which are parsimony-informative (Figure 3). One reason for this could be that isolate R265 was collected in BC while the majority of other isolates were from the PNW. However, B8577 is an environmental isolate from BC so it might be expected to be more closely related to R265, but this was not the case in this analysis. Another explanation could be that isolate R265 is also temporally separated from the other isolates, however this only represents a six year difference. An alternative, and we believe more plausible, explanation for this divergence could be the presence of sequencing errors in the reference strain, R265, which was sequenced with an average coverage of 6.5×, using Sanger sequencing. [16]. Sequencing errors in the reference would have limited impact on the diversity among the VGIIb and VGIIc clades in this analysis. As only shared orthologous SNPs were used in the analyses, a lower average read depth in a given WGST data set would decrease the shared portion of the reference genome interrogated for SNPs in each analysis, potentially decreasing the number of SNPs found among any of the groups. The lowest percent reference genome coverage in the VGIIa data set was greater (96.7%) than the lowest coverage found in the VGIIc data set (92.9%; Table 1).

While VGII subtypes had been identified in several collections globally distributed in Australia, Europe and South America [22]–[24], neither the VGIIa nor the VGIIc subtypes had been previously identified from any global collections. The novelty of the subtypes and their recent identification in the PNW are indications that these are recently emerged subtypes. The VGIIb subtype, which has been previously identified in other parts of the world, could have appeared in the PNW much earlier than VGIIa and VGIIc [7], [12], [14]. While the number of WGST SNPs identified among the VGIIa (after removing the R265 sequence from the analysis) and VGIIc isolates is similar to the number identified among the VGIIb isolates (Figure 3), this may simply be due to fewer VGIIb isolates being included in the analysis. There appears to be as much, if not more, genetic diversity among the four VGIIb isolates than any four of the VGIIa or VGIIc isolates as indicated by the relative branch lengths of the isolates. While further analysis with additional VGIIb isolates is necessary for confirmation, the data presented here is consistent with the current belief that the VGIIb clone radiated earlier than VGIIa or VGIIc clones, thus having more time to establish distinct sublineages.

Our study provides further evidence of the distinct nature of the *C. gattii* clonal subtypes causing disease in the PNW. WGST revealed definitively that the VGIIc subtype of *C. gattii*, the predominant causative agent of recent cryptococcal illness in Oregon [12], [13], is genetically distinct from both the VGIIa and VGIIb subtypes. Additionally, while MLST analysis shows that VGIIa and VGIIc are slightly more closely related to each other than to VGIIb, WGST analysis indicates that VGIIb and VGIIc are slightly more closely related to each other than to VGIIa. All three subtypes do appear to be equally genetically distinct from one another, as indicated by the nearly identical branch lengths for each of the subtype clades. As such, it seems unlikely that the VGIIb clone gave rise to the VGIIa and/or VGIIc clones, as has been previously suggested. WGST analysis of an expanded collection of VGII isolates, including analyzing patterns of shared diversity, is planned and may help to better characterize the relationships among these three important subtypes.

The VGI and VGIII isolates were included in this study as outgroups to anchor the analysis, but the genome data provide important information. The diversity (∼290,000 SNPs) seen among the VGI, VGII and VGIII molecular types is consistent with previous reports that assigned varietal status to these types [11], [16]. While this was not a goal of the current analysis, further analysis of the WGST data will help to delineate the genomic differences between the *C. gattii* molecular types and subtypes as well as differences in pathogenesis [5], [7], [11].

The comprehensive SNP data sets provided by WGS analysis will help facilitate the development of an accurate molecular clock, which will facilitate a better understanding of the evolutionary history of this organism. One of the most important aspects of this analysis is that we will now be able to individually delineate every *C. gattii* isolate collected. Large numbers of SNPs enable robust phylogenetic analyses, leading to more accurate phylogenetic reconstructions, especially in recombining organisms [15], [25]. The delineated phylogenetic relationships may allow us to link travel to exposure, map the spread of clonal lineages, and with the future development of a library of environmental isolates we may be able to delineate both the geographic and clonal origin of this emergence. We may eventually be able to link molecular subtypes as well as specific genotypes with pattern or severity of illness which would be of significant public health importance. SNP-based WGST phylogeny may not capture all small genetic differences due to sexual recombination that could lead to phenotypic changes. Detection of these types of changes would require more comprehensive genome sequence comparison to find structural variation such as rearrangements or insertion/deletion events. However, the fine-scale resolution of WGST has been shown to be a powerful and discriminatory molecular tool that can link epidemiologically related isolates in epidemiologic investigations [15], [26], [27].

Data generated from this study have established a framework from which computational analyses can be designed and conducted to better characterize the *C. gattii* and to provide robust tools for rapidly detecting emerging strains and efficiently responding to outbreaks.

## Supporting Information

Figure S1 **Phylogenetic analysis of WGST data from** ***C. gattii*** **,** ***C. grubii*** **and** ***C neoformans*** **isolates.** Maximum parsimony phylogenetic analysis was performed in MEGA4 on *C. gattii*, *C. neoformans* and *C. grubii* whole genome sequence data [19]. The R265 whole genome sequence was used as the reference for SNP discovery. The tree is rooted on the *C. grubii*/*C. neoformans* branch; one of 190 most parsimonious trees is shown. Bootstrap values less than 50% and for intra-VGIIa, VGIIb and VGIIc nodes are not shown. The numbers of SNPs included in the analysis is indicated (number of parsimony-informative SNPs in parentheses), as is the consistency index (CI) as calculated by MEGA4. While WGST analysis found unique genotypes for all isolates, they are not visible on this tree due to the large numbers of SNPs separating the VGII isolates from the other isolates.
(TIF)
