# Supplementary figures and images for: Whole Genome Sequence Analysis of Cryptococcus gattii from the Pacific Northwest Reveals Unexpected Diversity
Source: PLoS One. 2011 Dec 7;6(12):e28550. doi: 10.1371/journal.pone.0028550 (PMC3233577; doi:10.1371/journal.pone.0028550)

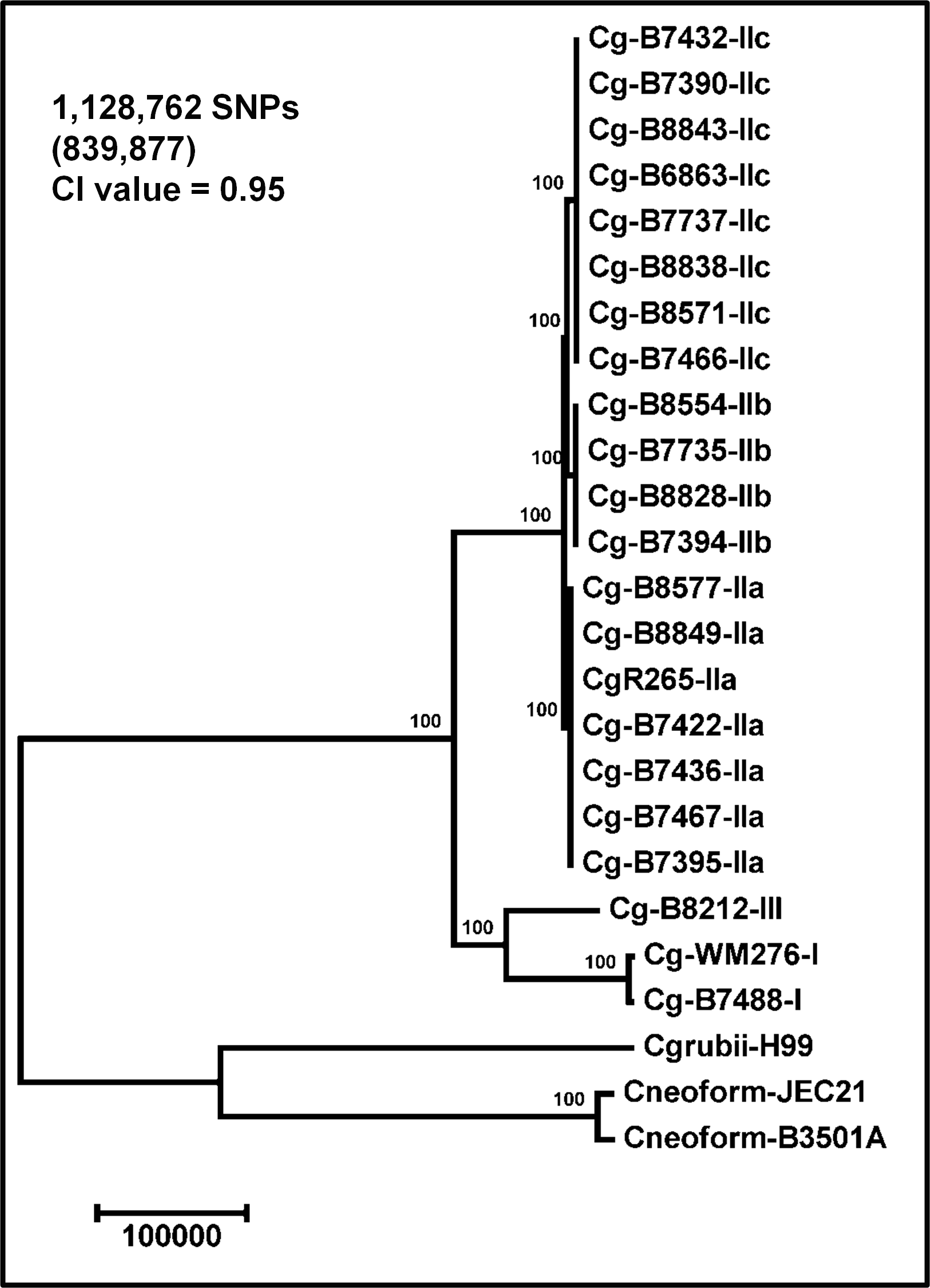

Supplement: Figure S1 — Phylogenetic analysis of WGST data from C. gattii , C. grubii and C neoformans isolates. Maximum parsimony phylogenetic analysis was performed in MEGA4 on C. gattii, C. neoformans and C. grubii whole genome sequence data [19]. The R265 whole genome sequence was used as the reference for SNP discovery. The tree is rooted on the C. grubii/C. neoformans branch; one of 190 most parsimonious trees is shown. Bootstrap values less than 50% and for intra-VGIIa, VGIIb and VGIIc nodes are not shown. The numbers of SNPs included in the analysis is indicated (number of parsimony-informative SNPs in parentheses), as is the consistency index (CI) as calculated by MEGA4. While WGST analysis found unique genotypes for all isolates, they are not visible on this tree due to the large numbers of SNPs separating the VGII isolates from the other isolates. (TIF) [file pone.0028550.s001.tif]
